# Exploration of the Delivery of Oncolytic Newcastle Disease Virus by Gelatin Methacryloyl Microneedles

**DOI:** 10.3390/ijms25042353

**Published:** 2024-02-16

**Authors:** Qiang Zhang, Jintong Na, Xiyu Liu, Jian He

**Affiliations:** State Key Laboratory of Targeting Oncology, National Center for International Research of Bio-Targeting Theranostics, Guangxi Key Laboratory of Bio-Targeting Theranostics, Collaborative Innovation Center for Targeting Tumor Diagnosis and Therapy, Guangxi Medical University, Nanning 530021, China; zhangqiang@sr.gxmu.edu.cn (Q.Z.); najtong@163.com (J.N.)

**Keywords:** Newcastle disease virus, gelatin methacryloyl, microneedles, hydrogel, oncolytic virus delivery

## Abstract

Oncolytic Newcastle disease virus is a new type of cancer immunotherapy drug. This paper proposes a scheme for delivering oncolytic viruses using hydrogel microneedles. Gelatin methacryloyl (GelMA) was synthesized by chemical grafting, and GelMA microneedles encapsulating oncolytic Newcastle disease virus (NDV) were prepared by micro-molding and photocrosslinking. The release and expression of NDV were tested by immunofluorescence and hemagglutination experiments. The experiments proved that GelMA was successfully synthesized and had hydrogel characteristics. NDV was evenly dispersed in the allantoic fluid without agglomeration, showing a characteristic virus morphology. NDV particle size was 257.4 ± 1.4 nm, zeta potential was −13.8 ± 0.5 mV, virus titer TCID50 was 10^7.5^/mL, and PFU was 2 × 10^7^/mL, which had a selective killing effect on human liver cancer cells in a dose and time-dependent manner. The NDV@GelMA microneedles were arranged in an orderly cone array, with uniform height and complete needle shape. The distribution of virus-like particles was observed on the surface. GelMA microneedles could successfully penetrate 5% agarose gel and nude mouse skin. Optimal preparation conditions were freeze-drying. We successfully prepared GelMA hydrogel microneedles containing NDV, which could effectively encapsulate NDV but did not detect the release of NDV.

## 1. Introduction

The incidence and mortality caused by tumors are increasing worldwide, and new drugs for tumor-targeted therapy are continuously being developed. Oncolytic viruses are a class of new drugs for cancer treatment. Oncolytic viruses can target tumor cells, and replicate and proliferate in tumor cells, leading to tumor cell lysis and death. The immunogenic death of tumor cells will further release tumor-associated antigens, pathogen-associated molecular patterns, danger-associated molecular patterns and cytokines, promote the maturation and activation of dendritic cells, activating and causing proliferation of cytotoxic T lymphocytes, resulting in a systemic anti-tumor immune response. Oncolytic viruses include Coxsackie virus, poliovirus, measles virus, and Newcastle disease virus (NDV) [1,2]. 

In this study, the Newcastle disease virus was used for oncolytic treatment. Newcastle disease virus is a single-stranded negative-sense RNA avian paramyxovirus with a spiral capsid and envelope between 100–500 nm in particle size [3]. It infects cells through cell membrane fusion or the direct endocytosis of the virus and replicates in the cytoplasm, so the infected cells are not affected by the insertion mutation and have good biosafety [4]. Oncolytic Newcastle disease virus is a tumor targeting virus, which can inhibit the growth of a variety of tumor cells. Oncolytic viruses have good biological safety and are not lethal to normal cells. In order to verify the release and expression of Newcastle disease virus, HepG2 and L02 were used in the cell experiments. HepG2 is a human liver cancer cell line commonly used in the laboratory [5], and L02 is a human normal hepatocyte line [6,7]. The cytotoxic effects of NDV on the human hepatoma cell line HepG2 [8,9] and human normal liver cell line L02 [10,11] were reported in the literature, which confirmed the tumor specificity and biological safety of NDV.

The current delivery methods of oncolytic viruses mainly include intravenous injection [12], intratumoral injection [13,14] and peritumoral injection [15]. Intravenous delivery of the virus presents several challenges such as easy virus clearance by the reticuloendothelial system, immune cells and neutralizing antibodies, poor tumor targeting, and serious damage to systemic organs [16]. Many oncolytic viruses cannot reach brain tissue through blood circulation to exert therapeutic effects because of the blood–brain barrier [17]. Problems associated with intratumoral and peritumoral injections include dense interstitial tissue in the tumor and poor lymphatic drainage, limiting the effective penetration and even distribution of the virus throughout the tumor. Previous studies have demonstrated that the delivery of oncolytic viruses via the tumor in situ hydrogel can protect the oncolytic virus from degradation, improve the tumor-specific targeting of the oncolytic virus, and allow the sustained release of the virus [18,19,20].

Hydrogels are biomaterials with hydrophilic three-dimensional grid structures, formed through the physical or chemical cross-linking of polymers. Hydrogels can protect drugs from premature degradation maintaining their biological activity and, after photocrosslinking, the hydrogels provide the slow-release of the drugs, enhancing the concentration and action time of drugs in tumors, while avoiding the side effects caused by the sudden release of the drugs [21].

Microneedles are microarray structures composed of triangular pyramid or quadrangular pyramid needle tips with a height of 100–1000 μm, which are a minimally invasive and painless transdermal drug delivery vehicle [22]. Microneedles play a targeted anti-tumor role, which can effectively penetrate a local tumor and uniformly release drugs, increase the penetration depth and action area of drugs, and significantly improve the delivery efficiency of drugs in tumor tissues [23]. Microneedles encapsulating different tumor drugs, such as chemotherapy drugs [24], immune cells [25], tumor vaccines [26], and photosensitizers [27], are commonly used in various cancer treatments, such as chemotherapy, immunotherapy, photodynamic therapy, and photothermal therapy.

Methacrylic anhydride is often used to chemically modify gelatin [28,29], chitosan [30,31], silk fibroin [32] and other polymers to generate photo cross-linkable hydrogels. In this paper, photo cross-linkable gelatin methacryloyl was prepared by chemical modification. Gelatin is a derivative of collagen with good biocompatibility. At the same time, because gelatin can be degraded by matrix metalloproteinases (MMPs) such as collagenases [33], which are enzymes that are overexpressed in almost all human cancers [34], GelMA hydrogels are often used as tumor collagenase-responsive hydrogels.

The advantages of GelMA microneedles in drug delivery are mainly reflected in their excellent biocompatibility, puncture performance and ability to achieve the slow-release of drugs. GelMA is a type of hydrogel material that can be photocrosslinked. GelMA microneedles with different release rates can be prepared by controlling the degree of the crosslinking of the gel. Current research has shown that GelMA microneedles can be used to deliver a variety of drugs including bioactive drugs, such as plasmid DNA (pDNA) [35], Epidermal growth factor (EGF) [36] and platelet rich plasma (PRP) [37]. GelMA can also be used as the matrix of frozen microneedles to deliver live bacteria [38]. At present, GelMA microneedles can also be used to deliver live viruses. In one study, GelMA microneedles were loaded with an adeno-associated virus expressing vascular endothelial growth factor (AAV-VEGF). The porous structure after UV crosslinking captured the AV-VEGF in the microneedle network and the microneedles were inserted into the cerebral cortex providing a sustained and controlled release of AAV-VEGF under the action of enzymatic degradation. It was demonstrated for the first time that GelMA microneedles loaded with AAV-VEGF can promote angiogenesis and neurogenesis after ischemic stroke and promote the recovery of corresponding functions [39].

The application of microneedles in virus delivery is mainly reflected in the transdermal inoculation of viral vaccines. Research has been conducted on the application of polylactic acid coated microneedles coated with live dengue virus [40] and live vaccinia virus [41] in vaccine delivery. The results have shown that the encapsulated virus can be effectively released into the skin tissue and effectively induce an immune response. The application of virus loaded microneedles in tumor therapy is mainly aimed at tumor immunotherapy, which can activate the immune system by delivering the virus and change the immunosuppressive environment of the tumor. A good tumor inhibitory effect was reported using cowpea mosaic virus nanoparticles delivered with soluble polyvinylpyrrolidone (PVP) [42]. microneedles However, the application of microneedles encapsulating oncolytic virus in tumor immunotherapy has not been previously reported.

**Figure 1:**
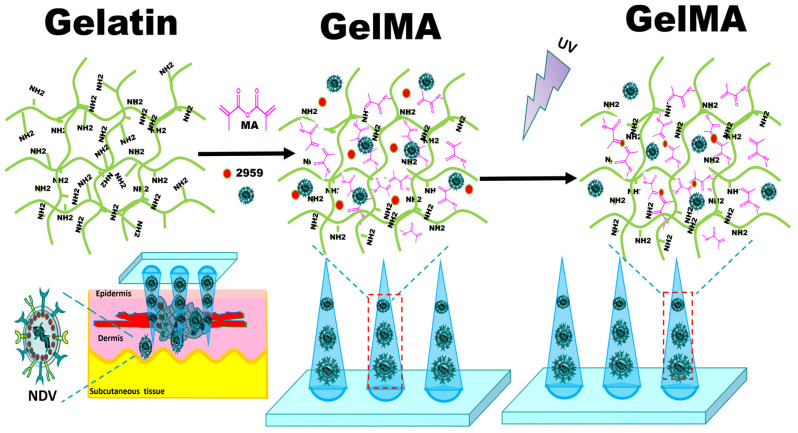
Schematic diagram of the preparation of GelMA microneedles encapsulating Newcastle disease virus by micro-molding and photocrosslinking.

In this study, we used degradable biocompatible GelMA photo crosslinked hydrogel as the matrix material of the microneedles, and mixed the hydrogel solution with an oncolytic Newcastle disease virus allantoic solution by vortex. We prepared NDV-loaded GelMA microneedles for the local delivery of the oncolytic Newcastle disease virus in tumors by using the procedures of micro-molding, photocrosslinking, and freeze-drying. The schematic diagram is shown in Figure 1.

## 2. Results

### 2.1. NMR-H_1_ Spectra of GelMA and Gelatin

**Figure 2:**
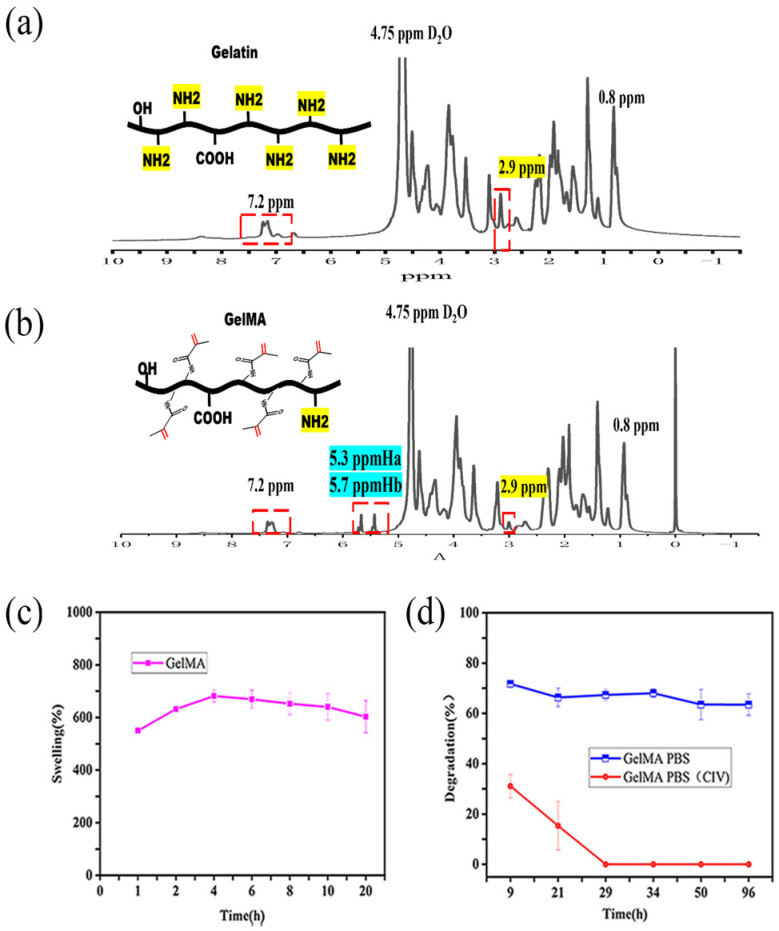
Characterization diagram of GelMA. (**a**) The NMR-H_1_ of gelatin. (**b**) The NMR-H_1_ of GelMA. (**c**) The swelling curve of GelMA in PBS. (**d**) The degradation curve of GelMA in PBS and PBS containing collagenase.

The peak at 2.9 ppm was the characteristic peak of the gelatin amino group, because the amino group in gelatin was amidated by methacrylic anhydride (MA), the height of the peak at 2.9 ppm significantly decreased, and because of the modification of MA, where the methacrylic anhydride -H2C=C(CH3)- was, there was an obvious double peak at δ = 5.3 ppm and δ = 5.7 ppm, the changes in the characteristic peaks indicated that MA was successfully modified on the gelatin molecules. The GelMA peak and gelatin peak at 7.2 ppm were similar, this corresponds to the characteristic peak of protein and does not participate in chemical reactions. Furthermore, 0.8 ppm was also a peak that does not participate in the chemical reaction, and 4.75 ppm was the characteristic peak of deuterated water (D_2_O) (Figure 2a,b). The degree of the substitution of methacrylic anhydride was measured to be above 20%, meeting the UV crosslinking conditions.

### 2.2. GelMA Swelling Rate and Degradation Rate Curve

The cuboid-shaped GelMA hydrogel samples containing 20% GelMA (size: 5 mm × 5 mm × 4 mm) were prepared by drying at 37 °C for 12 h. After approximately 4 h the synthesized GelMA hydrogel reached a swelling equilibrium in PBS, indicating that the synthesized GelMA hydrogel had the characteristics of rapid water absorption and swelling. The swelling percentage decreased slightly in the later stage, which might be related to the loss of mass during the weighing process of the hydrogel (Figure 2c). The quality of the synthesized GelMA hydrogel was almost constant in PBS for four days, indicating that GelMA hydrogel has good stability, and was not easy to hydrolyze under physiological conditions. The GelMA hydrogel was completely degraded in PBS containing 2 U/mL CIV collagenase within 24 h, confirming that the GelMA hydrogel was collagenase-responsive (Figure 2d).

### 2.3. NDV Transmission Electron Microscope

**Figure 3:**
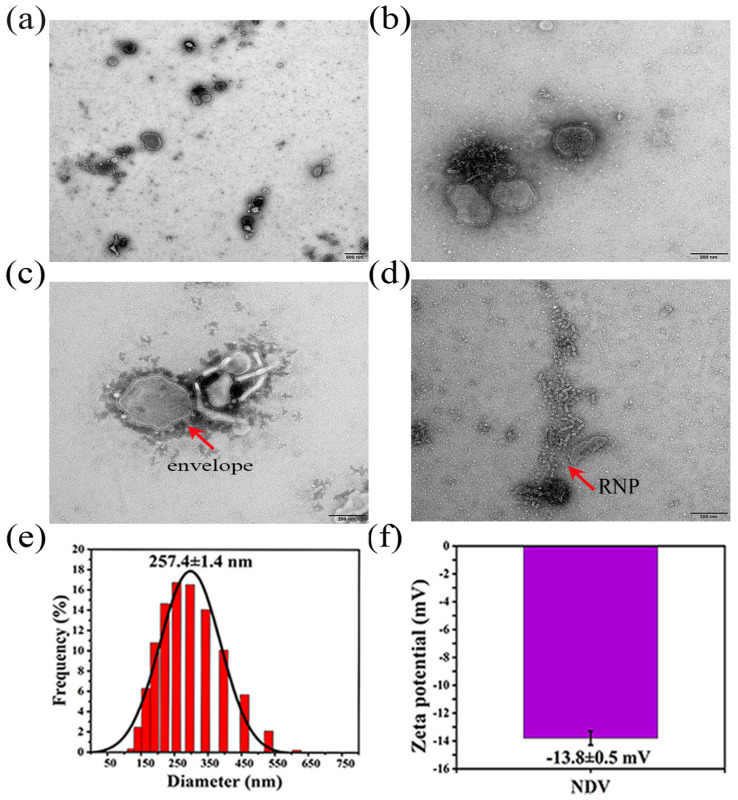
Characterization diagram of NDV. (**a**) The dispersion degree of NDV in the allantoic fluid, taken by scanning electron microscopy (scale bar 500 nm). (**b**) The morphology of NDV, taken by scanning electron microscopy (scale bar 200 nm). (**c**) The characteristic envelope of NDV, taken by scanning electron microscopy (scale bar 200 nm). (**d**) The aggregated ribonucleoprotein (RNP) of NDV, taken by scanning electron microscopy (scale bar 200 nm). (**e**) The particle size distribution of NDV. (**f**) The zeta potential diagram of NDV.

TEM images showed that Newcastle disease virus was evenly dispersed in the allantoic fluid without aggregation (Figure 3a). The virus particles were polymorphic, with round, oval, and irregular shapes. There was an obvious envelope on the surface of the virus and there were evenly distributed spike structures outside the envelope (Figure 3b,c). Aggregation of the ribonucleoprotein in the allantoic fluid was observed (Figure 3d).

### 2.4. NDV Particle Size and Zeta Potential

The NDV particle size was measured three times, and the measurements were 255.9 nm, 257.8 nm, and 258.6 nm with an average particle size and standard deviation of 257.4 ± 1.4 nm, which were consistent with the results of the transmission electron microscopy (Figure 3e). The polydispersity index (PDI) of the NDV, measured three times, was 0.172, 0.194, and 0.196. These values were all less than 0.2, indicating that the NDV virus particles were scattered in the allantoic fluid without agglomeration, which was consistent with the results of the transmission electron microscopy. The potential values of the NDV measured three times were −13.3 mV, −13.8 mV, and −14.3 mV, and the average potential and standard deviation were −13.8 ± 0.5 mV (Figure 3f).

### 2.5. TCID50 Immunofluorescence Assay of NDV

**Figure 4:**
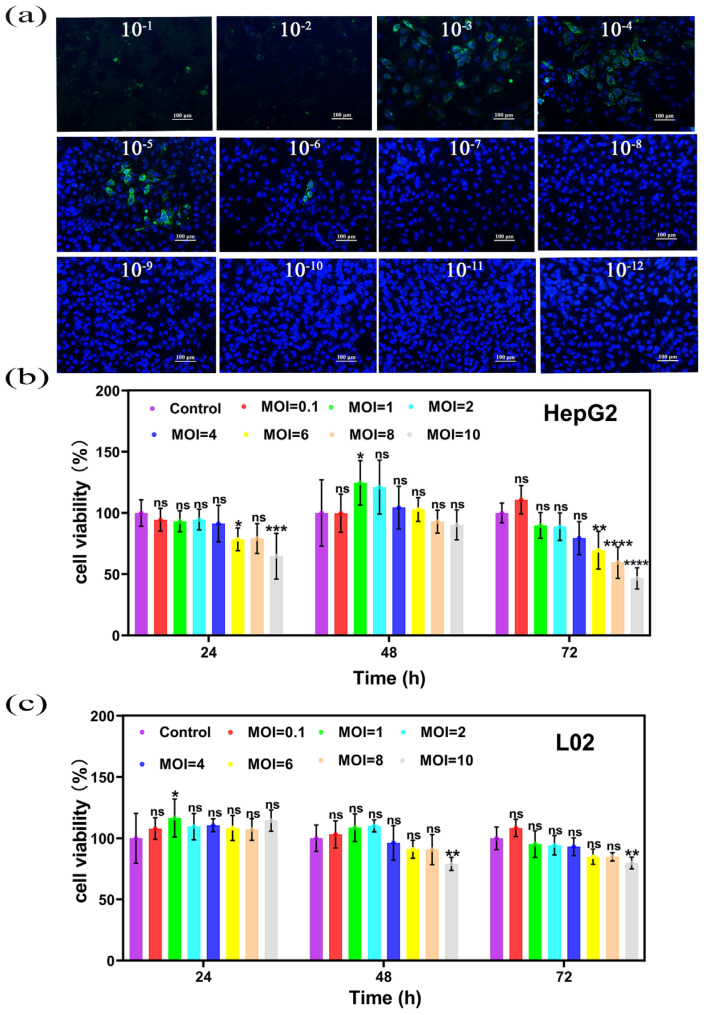
(**a**) The immunofluorescence diagram of TCID50 determination of NDV, with a scale of 100 nm. (**b**) The cell survival rate of HepG2 cells at 24 h, 48 h and 72 h after NDV treatment. (**c**) The cell survival rate of L02 cells at 24 h, 48 h and 72 h after NDV treatment. Data were shown as mean ± SD (*n* = 3) and analyzed using the one-way ANOVA test. Significances were presented by * *p* < 0.05, ** *p* < 0.01, *** *p* < 0.001, **** *p* < 0.0001, ns: not significant.

When NDV dilution was 10^−1^–10^−2^, there was no blue fluorescence of the nucleus nor green fluorescence of the cytoplasm, and there were few cells left at the bottom of the dish in the bright field view, indicating that the concentration of the 10^−1^–10^−2^ viruses is too high, the cytopathic effect was serious, and the cells had fallen off the dish after death. When NDV dilution was 10^−3^–10^−6^, obvious green fluorescence could be observed in the cytoplasm of the cells, and with NDV dose reduction, the number of positive cells with green fluorescence decreased, and only two or three positive cells per field could be observed in the 10^−6^ dilution. When NDV dilution was 10^−7^–10^−11^, only the blue fluorescence signal of the nucleus could be observed, and no green fluorescence-positive cells could be detected, indicating that NDV diluents with subsequent dilutions could not express the NDV antigen in cells (Figure 4a). The number of NDV positive wells was eight in different dilutions from 10^−1^ to 10^−6^.The number of NDV positive wells was zero in different dilutions from 10^−7^ to the negative control. Pores with lesions in 50% of cells were located between 10^−6^–10^−7^, the distance ratio was −0.5, TCID50 = 10^7.5^/mL, PFU = 0.7 × TCID50 = 0.7 × 10^7.5^/mL ≈ 2 × 10^7^/mL, so the PFU of NDV was 2 × 10^7^ PFU/mL (Table S1).

### 2.6. CCK-8 Detection of NDV Killing HepG2 and L02

The survival rate of HepG2 cells after NDV treatment showed that NDV had an obvious killing effect on HepG2 cells in a concentration and time-dependent manner. With the increase in the virus dose and the extension of the action time, the survival rate of HepG2 cells decreased (Figure 4b). The cell survival rate of L02 cells after NDV treatment showed that there was no significant difference in the killing effect on L02 among the low-dose groups with MOI < 10,while in the high-dose groups with MOI = 10, significant killing began at 48 h (Figure 4c). Based on the above results, it was concluded that no significant killing of HepG2 and L02 occurred at any dose groups with MOI < 6 within 72 h. There was significant killing of HepG2 and L02 at the same time at the dose groups with MOI = 10, but the killing rate of HepG2 was higher. There was an obvious killing effect on HepG2 but no obvious effect on L02 at the dose groups with MOI between 6–10 within 72 h. It was observed that the killing effect of MOI = 8 on HepG2 was stronger than that of MOI = 6. Therefore, MOI = 8 was selected as the NDV dose for subsequent experiments.

### 2.7. SEM Images of GelMA Microneedles with Different Contents and GelMA Microneedles Encapsulating NDV

**Figure 5:**
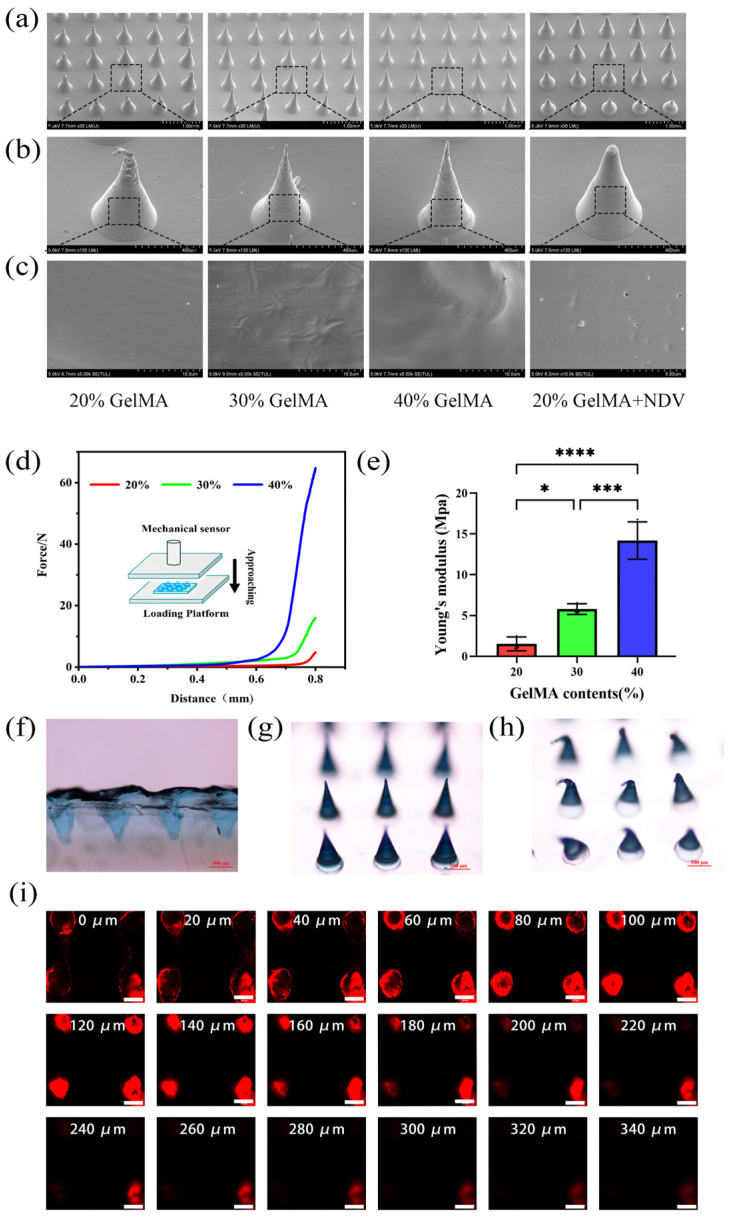
Characterization diagram of GelMA microneedles. (**a**) The SEM image of microneedle array, with a scale of 1 mm. (**b**) The SEM enlarged image of microneedle tip (scale bar 400 μm). (**c**) The SEM enlarged image of the surface of the microneedle tip with different contents and GelMA microneedles encapsulating NDV (scale bar 10 μm and 5 μm, respectively). The particles indicated by the red arrow are virus like particles. (**d**) The stress–strain curves of GelMA microneedles with different contents. (**e**) The Young’s modulus plots of GelMA microneedles with different contents. (**f**) The cross-sectional view of agarose gel after GelMA microneedles puncture (scale bar 500 μm). (**g**) The morphology of the microneedle tip before puncture (scale bar 500 μm). (**h**) The morphology of the microneedle tip after puncture (scale bar 500 μm). (**i**) The confocal fluorescent layer scan of the nude mice skin punctured with GelMA microneedles (scale bar 200 μm). Data were shown as mean ± SD (*n* = 3) and analyzed using the one-way ANOVA test. Significance was presented as * *p* < 0.05, *** *p* < 0.001, **** *p* < 0.0001.

Figure 5 shows GelMA microneedles containing 20%, 30% and 40% GelMA, and shows GelMA microneedles containing 20% GelMA and NDV. As shown in the first row, the array of microneedles is arranged neatly (scale: 1 mm) (Figure 5a). As shown in the second row, the single needle is tapered, and the needle tip structure is complete, so as to ensure that there is a sufficient mechanical basis for percutaneous penetration (scale: 400 μm) (Figure 5b). As shown in the third row, the surface of microneedles with different GelMA contents is smooth without a porous structure (scale: 10 μm) Virus-like particles (shown by the red arrow) are distributed on the surface of GelMA microneedles encapsulating NDV (scale: 5 μm) (Figure 5c).

### 2.8. Stress–Strain Curves of GelMA Microneedles with Different Contents

The GelMA microneedles encapsulating NDV were prepared by drying at 37 °C for 12 h. Figure 5 shows the stress–strain curves of GelMA microneedles containing 20%, 30% and 40% GelMA from right to left, respectively. With the increase in GelMA content, the stress–strain curve gradually shifted to the left and, under the same stress, the strain gradually decreased (Figure 5d) and Young’s modulus gradually increased (Figure 5e), indicating a gradual increase in mechanical strength.

### 2.9. Agarose and Mouse Skin Puncture Test

The GelMA microneedles containing 20% GelMA were prepared by drying at 37 °C for 12 h. As shown in Figure 4, the microneedles were used to puncture agarose and nude mouse skin. It was observed that GelMA microneedles containing 20% GelMA effectively penetrated 5% agarose gel (Figure 5f). And the needle tip deformed after puncture, the microneedle tip before puncture was Photographed by inverted microscope (Figure 5g), the microneedle tip after puncture was Photographed by inverted microscope (Figure 5h). It was also observed that GelMA microneedles can reach into skin tissue at a depth of 320 μm, indicating that GelMA hydrogel microneedles can effectively puncture the skin of nude mice and form microchannels in the skin to allow the release of drugs (Figure 5i).

### 2.10. Immunofluorescence of NDV and NDV-Loaded GelMA Microneedles

The GelMA microneedles encapsulating NDV were prepared by drying at 37 °C for 12 h. As can be seen from the fluorescence, NDV was not detected in the immunofluorescence diagram of the release solution at all time points. The positive control group was NDV allantoic fluid stock solution, with a large amount of green fluorescence. The negative control group was PBS, without green fluorescence (scale bar 100 μm) (Figure 5a).

### 2.11. Hemagglutination Titers of NDV and NDV-Loaded Microneedles

**Figure 6:**
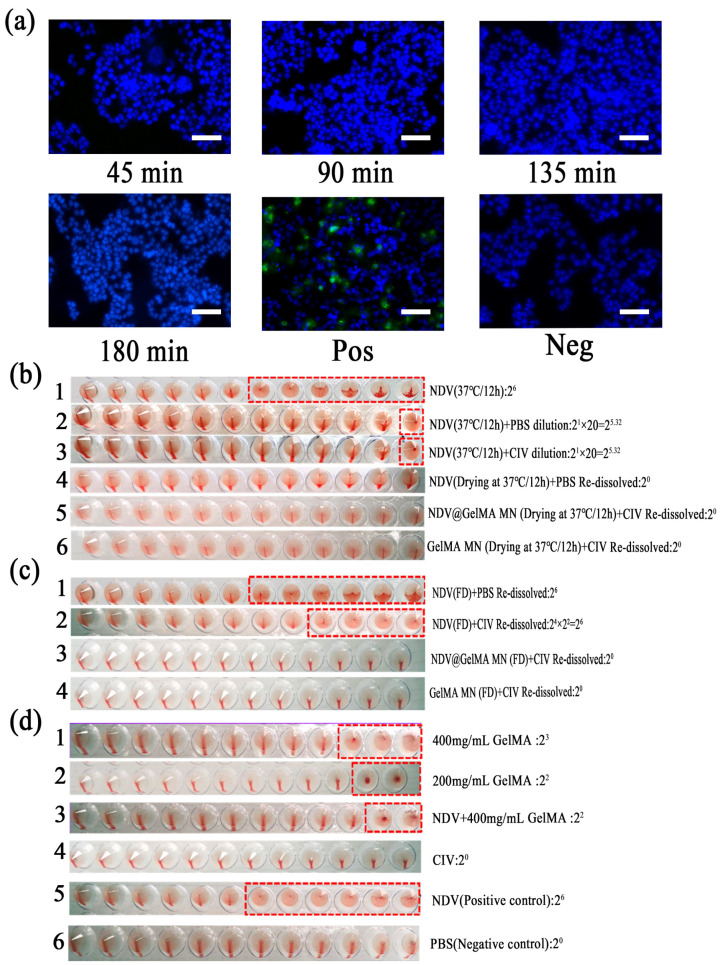
Characterization diagram of GelMA microneedles encapsulating NDV. (**a**) The immunofluorescence diagram of the release solution of GelMA microneedles encapsulating NDV at 45 min, 90 min, 135 min, and 180 min. (**b**–**d**) The hemagglutination diagram of different sample solutions. The hemagglutination result shown in the red box is positive.

NDV allantoic fluid was still liquid after being sealed at 37 ° C for 12 h, the hemagglutination titer was 2^6^ (Figure 6(b1)). NDV allantoic fluid was still liquid after being sealed at 37 °C for 12 h, and after being diluted with PBS or collagenase, the hemagglutination titer were both approximately 2^6^ (Figure 6(b2,b3)). NDV allantoic fluid became solid after being dried at 37 °C for 12 h, and after being re-dissolved with PBS in equal proportion, the hemagglutination titer was 2^0^ (Figure 6(b4)). GelMA microneedles encapsulating NDV and GelMA microneedles were prepared by constant temperature drying at 37 °C for 12 h, and after being re-dissolved by collagenase at 37 °C for 30 min, the microneedles were completely dissolved, the hemagglutination titers of the solution were both 2^0^ (Figure 6(b5,b6)).

NDV allantoic fluid became solid after being freeze-drying for 12 h, and after being re-dissolved with PBS or collagenase solution in equal proportion, the hemagglutination titer were both 2^6^ (Figure 6(c1,c2)). GelMA microneedles encapsulating NDV and GelMA microneedles were prepared by freeze-drying for 12 h, and after being re-dissolved by collagenase at 37 °C for 30 min, the microneedles were completely dissolved, the hemagglutination titers of the solution were both 2^0^ (Figure 6(c3,c4)).

The hemagglutination titer of 400 mg/mL GelMA solution was 2^3^ (Figure 6(d1)). The hemagglutination titer of 200 mg/mL GelMA solution was 2^2^ (Figure 6(d2)). The hemagglutination titer of 400 mg/mL GelMA solution mixed with the NDV allantoic fluid in equal proportion was 2^2^ (Figure 6(d3)). The hemagglutination titer of the collagenase solution was 2^0^ (Figure 6(d4)). The hemagglutination titer of NDV allantoic fluid stored at −80 °C was 2^6^, which was used as the positive control of the experiment (Figure 6(d5)). The hemagglutination titer of PBS was 2^0^, which was used as the negative control of the experiment (Figure 6(d6)).

## 3. Discussion

### 3.1. NMR-H^1^ Spectra of GelMA and Gelatin

At present, there are two commonly used methods to determine the degree of substitution of GelMA. One is the trinitrobenzene sulfonic acid (TNBS) method [43], the other is the nuclear magnetic resonance hydrogen spectroscopy (NMR-H^1^) method [44]. MA substitution degree is a parameter to measure the UV crosslinking strength of GelMA. Under the same UV irradiation, the higher the substitution degree, the stronger the UV crosslinking strength [45].

### 3.2. GelMA Swelling Rate and Degradation Rate Curve

The hydrolysis mechanism of GelMA is that water molecules are added to the polymer skeleton, resulting in the breaking of ester bonds on the polymer skeleton [46]. The degradation mechanism of GelMA is caused by the breaking of the amide bonds in the gel network [47]. The hydrolysis or degradation caused by the breaking of ester bonds and amide bonds is non-specific and slow, while enzymatic degradation is the use of enzymes with a specific sequence to degrade the sensitive sequence in GelMA, so that GelMA can be degraded specifically and rapidly [48].

GelMA is a type of gelatin derived material modified by methacrylic anhydride. Because it contains matrix metalloproteinases (MMPs) sensitive motifs, it can be hydrolyzed by MMPs. Under physiological conditions, GelMA is hardly degraded due to less secretion of MMPs. In tumor tissues, the GelMA degradation rate increases and drug release increases due to significantly increased secretion of MMPs. Gelatinase, also known as matrix metalloproteinases-2 (MMPs-2) or type IV collagenase, is the enzyme with the highest ability to destroy collagen (mainly type IV collagen) and can specifically hydrolyze gelatin [48,49]. Therefore, type IV collagenase is used in this paper. These experiments used an enzyme concentration of 2U/mL in GelMA enzymatic degradation based on the existing literature [35,36].

In this paper, in order to study the swelling properties of GelMA, we used gel blocks instead of microneedles. The reason for this choice is that gel blocks can be produced in batches and at one time, saving on experimental time, although the overall preparation conditions are the same as those for microneedles. When investigating the morphology, mechanical properties, and puncture properties of GelMA microneedles, microneedles should be used [36,50,51].

### 3.3. NDV Transmission Electron Microscope and Particle Size and Zeta Potential

Figure 3 shows that the virus is typical in shape, evenly dispersed, with a potential of about −13.8 mv, and a particle size of about 257.4 nm. The negative charge of the virus is conducive to its combination with the gel through electrostatic adsorption, and the appropriate particle size is conducive to its effective release from the gel pore [20].

### 3.4. TCID50 Immunofluorescence Assay of NDV

TCID50 is a commonly used virus titer test, which can quantify the virus and help to determine the number of viruses encapsulated and released in subsequent experiments. TCID50 results were transformed into PFU, which can be used for virus quantification in subsequent cell experiments [52,53,54].

### 3.5. CCK-8 Detection of NDV Killing HepG2 and L02

Studies have reported that NDV can specifically recognize the sialic acid receptor on the surface of tumor cells, thereby specifically infecting tumor cells, and because of the abnormal interferon pathway in tumor cells, its ability to clear the virus is weakened, which ultimately leads to the replication and proliferation of NDV in tumor cells, cracking tumor cells and activating the cellular immune response to jointly inhibit the growth of tumor cells. For normal cells, such as L02 cells, it will not cause damage [4,15,55,56].

### 3.6. SEM Images of GelMA Microneedles with Different Contents and GelMA Microneedles Encapsulating NDV

The surface morphology, gel pore size and NDV distribution of GelMA microneedles with different contents and NDV loaded GelMA microneedles were characterized by scanning electron microscopy (SEM).

### 3.7. Stress–Strain Curves of GelMA Microneedles with Different Contents

Studies using PEGDA as the matrix material for microneedles have shown that the mechanical strength of microneedles increases with the increase in PEGDA content. Due to the brittleness of the PEGDA material, a fracture peak appears in the stress–strain curve [57]. There are also studies using cross-linked HA as the material for microneedles, with fractures occurring at around 200 um. The average single needle fracture force is 0.38N/needle, and no fracture peak appears in the stress–strain curve of this experiment [58], GelMA demonstrated good toughness and no fracture occurred over the entire testing process. It was only observed that the needle tip was bent under pressure, and the mechanical strength of the microneedle increased with the increase in GelMA content. Considering the need to add NDV bladder fluid in an equal proportion in the future, GelMA microneedles were prepared using a 20% content.

### 3.8. Agarose and Mouse Skin Puncture Test

Simulation of the tumor environment in vitro used 5% agarose gel. Firstly, because the hardness of 5% agarose gel is equivalent to that of skin tissue, it can simulate skin tissue in vitro to verify the penetration performance of microneedles. Secondly, the water in 5% agarose gel can simulate the matrix solution of the tumor microenvironment and can be used to study the microneedle dissolution process. The reason for choosing agarose as the gel material is that the preparation process for agarose gel is simple and rapid. The powder can be rapidly dissolved in liquid at 100 °C and rapidly transformed into gel at room temperature. Secondly, agarose gel is uniform and transparent, which is conducive to observing the puncture depth and the microneedle separation effect [48,59].

### 3.9. Immunofluorescence of NDV and NDV-Loaded GelMA Microneedles

Firstly, GelMA microneedles carrying oncolytic Newcastle disease virus were prepared using the 37 °C constant temperature drying method, and no virus expression was observed by immunofluorescence staining. Through a hemagglutination test, it was found that freeze-drying could effectively ensure the activity of virus. Therefore, the GelMA microneedles carrying oncolytic NDV were prepared using the freeze-drying method, but cellular immunofluorescence staining was not performed, which is a limitation of this experiment.

### 3.10. Hemagglutination Titers of NDV and NDV-Loaded Microneedles

The hemagglutination titer of NDV allantoic fluid is not affected by the following factors: firstly, it is not affected by temperature within 24 h liquid state (Figure 6(b1)). Secondly, it was not affected by collagenase and PBS dilution (Figure 6(b2,b3)).

Drying at 37 °C for 12 h can significantly reduce the hemagglutination titer of NDV, showing that 37 °C constant temperature drying was not suitable for the preparation of GelMA microneedles encapsulating NDV (Figure 6(b4–b6)). However, freeze drying did not affect the biological activity of NDV, suggesting that freeze drying could be used to prepare GelMA microneedles encapsulating NDV (Figure 6(c1,c2)). However, the microneedles encapsulating NDV prepared using the freeze-drying method did not detect the hemagglutination titer (Figure 6(c3,c4)). This result can be explained by the fact that the NDV had not been released in a short period of time (30 min). Although it can be seen from the macro that the hydrogel microneedles have been completely hydrolyzed by the enzymes, it is likely that the macro hydrogels are only broken into micron and nanometer sized hy-drogel particles encapsulating the virus, Therefore, the release of the virus still needs to be further demonstrated by extending the detection time and improving the detection environment.

The hemagglutination titer is proportional to the concentration of the GelMA solution. The reason for this result may be that GelMA hydrogel is sensitive to temperature, the sol-gel transition occurs at room temperature, and the formation of the gel network makes red blood cells gather together, leading to a false positive hemagglutination test. However, with the dilution of the gel, the gel network structure no longer had an aggregation effect on the red blood cells, and the red blood cells began to flow under the effect of gravity, and then the hemagglutination test was negative (Figure 6(d1,d2)).

The GelMA solution encapsulating NDV aggregates red blood cells and NDV in the gel grid at the same time. With the dilution of the gel, the hemagglutination test of the subsequent wells was still negative, and no NDV capable of agglutinating red blood cells was detected, which indirectly proved that the virus was not released from the gel (Figure 6(d3)).

In summary, we designed GelMA hydrogel microneedles that can be used for oncolytic NDV delivery, which has recently attracted increasing research attention in tumor immunotherapy. Strategies which have been shown to improve the delivery efficiency of the oncolytic NDV include PEGylated virus shells [28], polymer-coated viruses [29,30], biomineralized nanoparticles encapsulating oncolytic viruses [31], and drug delivery vehicles such as hydrogels [11,13,32,33,34,35]. However, because the biological activity of the oncolytic NDV is not easy to maintain in vitro, its drug delivery efficiency is generally low, and it is difficult to maintain the therapeutic dose of tumor tissue. To improve the efficacy of oncolytic NDV in cancer treatment, large doses, and repeated injections are required, which are associated with inconvenience and risks to patients. Therefore, it is important to develop a controllable delivery system that can maintain the biological activity of the oncolytic NDV and also target and continuously release the oncolytic NDV into tumor tissues.

To the best of our knowledge, there are few studies designed to deliver oncolytic NDV with GelMA hydrogel microneedles. Here, we prepared GelMA hydrogel microneedles that can effectively encapsulate the oncolytic NDV. The experiments show that GelMA hydrogel can effectively encapsulate the virus, and the stable gel network structure makes the hydrolysis of the encapsulated virus difficult in a physiological environment, thus playing a protective role for the encapsulated virus. Because GelMA hydrogel has excellent swelling performance and enzymatic degradation performance, it can ensure the locally responsive release of viruses in tumors. The synthesized GelMA hydrogel microneedles can penetrate 5% agarose and nude mouse skin, indicating that it has excellent skin puncture ability, ensuring penetration into the tumor to release the drugs. NDV has strong tumor targeting, can replicate and proliferate in tumor cells, selectively lyse tumor cells, and plays a role in cell killing. At the appropriate dose, it has no killing effect on human normal liver cell L02 cells and has a significant killing effect on human liver cancer HepG2 cells. Therefore, GelMA hydrogel microneedles loaded with NDV have excellent biocompatibility, safety, and tumor targeting.

However, the release of NDV was not detected in this study, which may be related to the following experimental factors: (1) Sample type: in this study, the release solution of GelMA microneedles encapsulating NDV prepared under 37 °C drying condition was detected, but the release solution of GelMA microneedles encapsulating NDV prepared under freeze-drying condition was not detected. (2) Detection method: in this study, only immunofluorescence and hemagglutination experiments were used to detect the release of NDV, which should be further verified by combining q-PCR, CCK-8, flow cytometry, and animal experiments. (3) Detection environment: the virus is a bioactive substance that needs to replicate in living cells to survive. Adverse survival conditions in vitro and excessive detection time are likely to cause virus inactivation in the detection process, which will interfere with the detection of virus release. (4) Detection time: although the hydrogel microneedles had been completely enzymatically hydrolyzed, it is likely that these broke from the macroscale hydrogel into microscale and nanoscale hydrogel particles with no release of the encapsulated virus. Therefore, subsequent release of the virus needs to be demonstrated by extending the detection time.

Several studies have investigated hydrogels loaded with oncolytic viruses, although most of these focused on the delivery of an oncolytic adenovirus. Previous studies used calcium alginate hydrogel microspheres [18], sodium alginate hydrogel [60], PUSMA hydrogel [61], and GHPA hydrogel [62] to encapsulate and deliver an oncolytic adenovirus, all of them could release virus locally in the tumor. Hydrogel delivery of the oncolytic NDV has also been achieved by using mercapto chitosan hydrogel with a continuous release of NDV [63]. However, the GelMA hydrogels in this study encapsulated NDV but the release of NDV was not detected. It should be considered that GelMA hydrogels may not be suitable for delivering NDV, and their gel properties at room temperature may also interfere with the detection of NDV release. Future studies should identify other types of hydrogels or soluble materials that can be used to encapsulate the virus in order to detect NDV release.

We propose to focus our future research on the following aspects. First, the use of tumor- responsive release elements to enhance the degradation of the gel in the local tumor and achieve drug release. Second, the GelMA hydrogel microneedles can be de-signed as a tumor local inflammatory niche that does not release the virus. Some stud-ies have immobilized the Zika virus in chitosan oligomer hydrogels, allowing immune cells to be recruited into the gel for activation, while avoiding damage caused by virus leakage [20]. By incorporating the cancer cells infected with the NDV virus into GelMA hydrogel microneedles, the virus proliferates in tumor cells and lyses tumor cells to re-lease tumor-associated antigens, forming a local inflammatory niche. Antigen-presenting cells are then recruited to the gel microneedles to be stimulated to activate and present antigens, further promoting the activation and proliferation of immune killer cells, thus activat-ing the systemic anti-tumor immune response.

## 4. Materials and Methods

### 4.1. Reagents, Viruses, Cell Lines, and Animals

Gelatin was purchased from Vetec (V900863, Saint Louis, America), 2-hydroxy-4’- (2-hydroxyethoxy) 2-methylphenylacetone was purchased from TCI (H1361, Tokyo, Japan), methacrylic anhydride was purchased from Sigma (276685, Saint Louis, America), collagenase type 4 was purchased from solarbio (C8160, Beijing, China), 5% BSA blocking solution was purchased from Solarbio (SW3015, Beijing, China). Anti-Newcastle Disease virus antibody was purchased from Abcam (ab34402, Cambridge, UK), and Goat Anti-Chicken IgY H&L (Alexa Fluor^®^ 488) was purchased from Abcam (ab150169, Cambridge, UK). The wild-type NDV (LaSota) attenuated strain was donated by Harbin Veterinary Research Institute and stored in the −80 °C ultra-low temperature refrigerator by our laboratory for future use. Suckling hamster kidney cells (BSR t7/5), human liver cancer cell line (HepG2), and human normal liver cells (L02) were purchased from ATCC and stored in our laboratory. These cells were cultured in DMEM medium containing 10% fetal bovine serum, 1% antibiotics and 4.5 g/L glucose at 37 °C and 5% CO2. The female nude mice (7 weeks old) were purchased from the Animal Experiment Center of Guangxi Medical University. All experiments adhered to the “Guidelines for the Care and Use of Laboratory Animals in China”. The Animal Investigation Ethics Committee of Guangxi Medical University reviewed and approved all animal handling and experimental projects.

### 4.2. Synthesis of GelMA

First, 10 g of gelatin was weighed and dissolved in 100 mL of PBS, and magnetically stirred at 300 rpm at 50 °C for 1 h to fully dissolve the gelatin. Using a syringe, 10 mL of methacrylic anhydride (MA) was aspirated and the flow rate of the syringe pump was set to 10 mL/h. MA was slowly and uniformly added to the 50 °C/600 rpm magnetically stirred gelatin solution within 1h, with continued stirring for 3 h after the injection stopped to fully react. After 3 h, 500 mL of PBS was added to the solution followed by magnetic stirring at 50 °C/600 rpm for 1 h to terminate the reaction. The reacted solution was poured into the dialysis bag, placed into a beaker containing 40 °C pre-heated ultrapure water, and dialyzed in a constant temperature magnetic stirring water bath at 40 °C/200 rpm for seven days, during which time the water was changed many times. After dialysis, the sample was centrifuged at 4000 rpm for 15 min, and the supernatant was transferred to a new centrifuge tube and freeze-dried for seven days to obtain GelMA, which was stored in the dark at −20 °C.

### 4.3. NMR Detection of GelMA

Gelatin (15.0 mg) and freeze-dried GelMA (15.0 mg) were weighed and placed into the EP tube. Next, 0.5 mL of deuterated water (D_2_O) was added to the EP tube, the samples was dissolved completely at 60 °C/30 min, and the solution concentration was 30 mg/mL. The liquid was then transferred to the detection tube, the sample was scanned at room temperature with Bruker Avance Ⅲ 600M nuclear magnetic resonance instrument to obtain the nuclear magnetic resonance hydrogen spectra of GelMA and Gelatin. Mestrenova software was used to normalize the peak areas of the hydrogen spectrum with the peak of 0.8 ppm that was not involved in the reaction as the standard peak. The peak areas of GelMA and gelatin at 2.9 ppm were A1 and A2, respectively. The substitution degree (DS) of MA was calculated using the substitution degree formula as follows:

DS (%) = 1 − (A1/A2) × 100%(1)

### 4.4. Determination of the Swelling Rate and Degradation Rate of GelMA

A collagenase solution of 2 U/mL was prepared. The cuboid-shaped GelMA hy-drogel samples containing 20% GelMA (size: 5 mm× 5 mm×4 mm) were prepared by drying at 37 °C for 12 h. Three parallel samples were set and weighed, and the initial dry weight (W0) were recorded. and samples were placed into the EP tube, then added 1 mL of PBS to the tube, placed the EP tube into a 37 °C 100 rpm constant temperature shaker and retrieved the samples at seven times points (1 h, 2 h, 4 h, 6 h, 8 h, 10 h, and 20 h), Absorbent paper was used to wipe off the water on the surface of samples and weighed. The wet weight of the samples at each time point were recorded as (W_wet_), and then the samples were placed in the original solution. Data collection continued and the swelling rate was calculated according to the following formula:

Swelling rate (%) = (W_wet_ − W_0_)/W_0_ × 100% (2)

The degradation rate of GelMA was determined by adding 1 mL of PBS without collagenase or PBS containing 2 U/mL CIV collagenase to the tube. The EP tube was placed into a 37 °C/100 rpm constant temperature shaker, the solution in the EP tube was discarded at six times points (9 h, 21 h, 29 h, 34 h, 50 h, and 96 h). The EP tube containing the sample was placed into a vacuum freeze-dryer for four hours to completely dry the sample. The sample was retrieved from the freeze dryer, its dry weight (W_dry_) was recorded and the degradation rate was calculated according to the following formula:

Degradation rate (%) = W_dry_/W_0_ × 100% (3)

### 4.5. Morphology Observation of NDV

A tungsten filament transmission electron microscope was used to observe the morphology of the negatively stained NDV. Firstly, the area containing multiple virus particles was selected for photographing to characterize the distribution of the virus in the allantoic fluid. Then, photos of individual or multiple virus particles were obtained to characterize the microstructure of virus envelope, spike, and nucleocapsid.

### 4.6. Determination of NDV Particle Size and Zeta Potential

Malvern nanoparticle size and zeta potential analyzer were used to measure the particle size and potential of NDV. After centrifuging NDV allantoic fluid at 4000 rpm for 5 min, 0.5 mL of supernatant of NDV allantoic fluid was added to the particle size dish, and the test was repeated three times. The medium was water, the refractive index was 1.590, and the absorbance was 0.010. After the test, the sample was recovered and added to the potential cell to test the potential.

### 4.7. TCID50 Determination of NDV

After resuscitation and passaging, BSR cells were plated and 100 μL of cell suspension containing 8000 cells were added to each well of the 96-well plate, and the percentage of cell fusion was observed after 24 h. When the cells were in good condition and the fusion rate exceeded 70%, NDV allantoic fluid was added to the cells. NDV allantoic fluid was prepared with different concentration gradients, 900 μL of DMEM containing 2% FBS was added to each well of the 12-well plate, and then 100 μL NDV allantoic fluid was added to the first well, mixed. Then, 100 μL of liquid from the first well was aspirated and added to the second well, diluted in sequence, and the concentration gradient was set to 10^−1^–10^−11^. The culture medium in the 96 well plate was discarded and 100 μL of NDV allantoic fluid with different dilutions was added to 96 well plates in turn, ranging from 10^−1^–10^−11^ from left to right. The last column of wells was set as the negative control without adding poison, and each column was set with 8 duplicate wells. The 96 well plate was placed into a 37 °C 5% CO_2_ incubator for 72 h and then removed for immunofluorescence staining and photography. Cells with green fluorescence were positive cells, a well containing positive cells was a positive well. The steps of NDV immunofluorescence staining are described in the Supplementary Materials. TCID50 was calculated according to the Reed-Muencha method and converted into PFU, and the MOI value was calculated according to the number of infected cells. The TCID50 calculation formula of NDV is shown in the Supplementary Materials.

### 4.8. Cell Viability Assay of NDV

HepG2 and L02 cells in the logarithmic growth phase were digested and collected, and the treatment method was the same as above, Briefly, 100 μL of cell suspension containing 5000 cells were added to each well of the 96-well plate, the plate was then placed in a 37 °C 5% CO_2_ incubator for 24 h, and six duplicate holes were set in each group. NDV solution was prepared with different concentration gradients, i.e., MOI = 0.1, 1, 2, 4, 6, 8, 10. First, the solution with MOI = 10 was prepared by adding 16 μL of NDV into 1mL DMEM containing 2% FBS, followed by dilution to a dose of MOI of 8, 6, 4, 2, 1, 0.1. The 96-well plate was taken out of the incubator, the medium of each well was aspirated and discarded. Each well was washed with PBS once and PBS was discarded. Then, 100 μL DMEM medium containing 2% FBS and different NDV doses were added. The 96-well plate was placed into a 37 °C 5% CO_2_ incubator, and removed at 24 h, 48 h, and 72 h, to aspirate and discard the medium of each well, washed each well with PBS once and discard PBS. DMEM containing 10% CCK-8 was added to each well without FBS and cultured for 0.5–2 h. The absorbance value (OD) of each well was detected with a microplate reader at 450 nm wavelength. The cell survival rate of the different groups was calculated according to the absorbance value. The cell survival rate was calculated according to the following formula:

Cell survival rate (%) = (A_sample_ − A_blank)_/(A_control_ − A_blank_) × 100%. (4)

### 4.9. Preparation of GelMA Microneedles with Different Contents and Preparation of GelMA Microneedles Encapsulating NDV

A cone-shaped microneedle array hydrophobic PDMS mold with needle height 1000 μm was selected with a needle tip diameter of 10 μm, a bottom diameter of 420 μm, center distance of 900 μm, number of arrays 15 × 15, groove size of 16 × 16 mm, and groove depth of 2 mm. GelMA solutions were prepared with different concentrations and 200 mg, 300 mg, and 400 mg GelMA were placed into brown bottles. Next, 1 mL photoinitiator solution (5% *w*/*v*) was added to each bottle and the bottles were placed under 75 °C constant temperature for 30 min to ensure that GelMA was fully dissolved. Then, 500 μL GelMA solutions with concentrations of 200 mg/mL, 300 mg/mL, and 400 mg/mL were placed in the mold and 500 μL GelMA solution with concentrations of 400 mg/mL to normal temperature and fully mixed with 500 μL NDV allantoic fluid, and then 500 μL of mixed liquid was added to the mold. The mold was placed into a centrifuge and centrifuged at 4000 rpm for 5 min at 37 °C. The mold was retrieved from the centrifuge and used the point light source of the UV curing instrument to irradiate 200 mg/mL, 300 mg/mL, 400 mg/mL GelMA solution for 10 s and to irradiate NDV@GelMA Solution for 30 s. The wavelength and power of UV were 365 nm and 375 mW/cm^2^, respectively. The UV crosslinked samples were dried in a 37 °C constant temperature incubator for 12 h. After 12 h, the sample was peeled from the mold and placed into a container containing desiccant for storage. Because the addition of NDV occupied the UV crosslinking site of GelMA and weakened the UV crosslinking effect, it was necessary to extend the UV irradiation time to enhance the crosslinking effect.

### 4.10. SEM Images of GelMA Microneedles with Different Contents and GelMA Microneedles Encapsulating NDV

The array, single needle tip and surface morphology of GelMA microneedles with different contents and GelMA microneedles encapsulating NDV were observed and photographed by field emission scanning electron microscope. NDV distribution and gel pore size on the surface of microneedle and the overall morphology of the microneedle were characterized.

### 4.11. Stress–Strain Curves of GelMA Microneedles with Different Contents

Zwick/Z2.5 universal material testing machine was used to detect the mechanical properties of GelMA microneedles with different contents. The GelMA microneedles with different contents were placed on the sample table, the needle tip facing the mechanical sensor, and the speed of the mechanical sensor moving down was set to 0.5 mm/min. The termination condition of the test was that the sensor moves down 0.8 mm after contacting the needle tip. The microneedle samples testing parameters were set to a height of 3 mm and a bottom area of 256 mm^2^, with three parallel samples set for each sample. The stress–strain curves of the samples were obtained and the young’s modulus diagram of GelMA microneedles with different contents were converted according to the slope of the linear region of the stress–strain curve.

### 4.12. Agarose Gel Puncture Experiment of GelMA Microneedles

Skin tissue was simulated using 5% agarose gel to verify the puncture performance of GelMA microneedles. Accurately weighed 0.5 g agarose powder was placed into a glass bottle and 10 mL PBS was added to the bottle, heated to 100 °C for 30 min to completely dissolve the agarose. During this time, the bottle was turned upside and down several times and 30 min later the solution was poured into a transparent cube with length, width and height of 3 cm. The solution was left standing and cooling for 10 min to obtain an agarose gel block for detecting microneedle puncture performance. A drop of blue dye solution was added into 1 mL GelMA solution and mixed well. Microneedles were prepared according to the above methods. The microneedles were inserted into a 5% agarose gel block, and vertically cut to obtain the cross-section. The cross-sectional view of the agarose gel inserted with microneedles and the morphological changes of microneedles before and after insertion were obtained using an upright microscope.

### 4.13. Mouse Skin Puncture Experiment of GelMA Microneedles

The skin of nude mice was used for the puncture experiment. The skin was obtained from 7-week-old female nude mice, and there was no damage and inflammation before the experiment. First, 100 μL 1 mg/mL rhodamine dye solution was added into 1 mL GelMA solution and mixed well. The microneedles were prepared according to the above methods. The microneedles were inserted into the skin of nude mice and the nude mice were then euthanized. The skin of the nude mice at the puncture site was cut off and placed on the glass slide with the epidermis upward. The skin of nude mice after puncture was photographed by confocal microscope to characterize the puncture ability of the microneedles and the depth of penetration into the skin. Images were taken vertically downward at 10 μm spacing layer by layer, from the epidermis to the interior, and the absence of fluorescent signal was taken as the termination condition.

### 4.14. NDV Release was Detected by Immunofluorescence

GelMA microneedles encapsulating NDV were prepared according to the above methods. The microneedles were placed into a EP tube, 2 mL of DMEM containing only 2% FBS without collagenase were added to the EP tube and the tube was placed into a 37 °C 100 rpm constant temperature shaker. Aspiration of 2 mL of release solution into a 5 mL new EP tube at 45 min, 90 min, 135 min, and 180 min was performed and 2 mL of new PBS was added to the EP tube for further testing. New EP tubes were labeled as R45 min, R90 min, R135 min, and R180 min and stored at 4 °C in a refrigerator for subsequent immunofluorescence staining. HepG2 cells in the logarithmic growth phase were digested and collected, and the treatment method was as described above. Briefly, 2 mL of cell suspension containing 2 × 10^5^ cells was added to each well of the 6-well plate and the plate was placed into a 37 °C 5%CO_2_ incubator for 24 h. When the cells were in good condition and the fusion rate exceeded 70%, 2 mL of NDV release solution was added at different time points to the first four wells. The virus solution with MOI = 10 was added to the fifth well as a positive control, v = MOI value × number of infected cells / PFU value = (10 × 20 × 10^4^) / (2 × 10^7^) mL = 100 uL. Then, 100 μL of NDV allantoic fluid was added to the fifth well. DMEM only containing 2% FBS was used as a negative control well. The plate was placed in 37 °C 5% CO_2_ incubator for 72 h. After 72 h, the 96-well plate was removed and immunofluorescence staining and photography were performed. Cells with green fluorescence were cells infected with NDV.

### 4.15. Hemagglutination Test of Different Sample Solutions

A 1% chicken red blood cell suspension and 10 U/mL collagenase solution were prepared. NDV allantoic fluid was always stored at −80 °C. Sample solutions were obtained and the hemagglutination titer was determined. If the experimental process involved diluting the sample, the hemagglutination titer was multiplied by the corresponding dilution multiple.

The sample preparation process in Figure 5a: 200 μL of NDV allantoic fluid sealed in an EP tube and placed at 37 °C for 12 h; 200 μL of NDV allantoic fluid sealed in an EP tube and placed at 37 °C for 12 h, followed by removal of 50 μL of the fluid which was then mixed with 950 μL of PBS; 200 μL of NDV allantoic fluid sealed in an EP tube and placed at 37 °C for 12 h, followed by removal of 50 μL of the above fluid which was then mixed with 950 μL of PBS containing collagenase. NDV allantoic fluid (200 μL) was added to a glass slide, placed at 37 °C for 12 h to form a solid, and then dissolved by adding 200 μL of PBS. NDV@GelMA microneedles were prepared by drying 500 μL of NDV and GelMA mixed solution at 37 °C for 12 h. Then, 5 mL of PBS-containing collagenase was added, and the microneedles were fully dissolved at 37 °C for 30 min. GelMA microneedles were prepared by drying 500 μL of 200 mg/mL GelMA solution at 37 °C for 12 h. Then, 5 mL of PBS containing collagenase was added, and the microneedles were fully dissolved at 37 °C for 30 min.

The sample preparation process in Figure 5b: 200 μL NDV allantoic fluid added to an EP tube, freeze-dried for 12 h to form a solid, and then dissolved by adding 200 μL of PBS. NDV allantoic fluid (200 μL) was added to an EP tube, freeze-dried for 12 h to form a solid, and then dissolved by adding 800 μL of PBS containing collagenase. NDV@GelMA microneedles were prepared by freeze-drying 500 μL of NDV and GelMA mixed solution at 37 °C for 12 h. Then, 5 mL of PBS containing collagenase was added, and the microneedles were fully dissolved at 37 °C for 30 min. GelMA microneedles were prepared by freeze-drying 500 μL of 200 mg/mL GelMA solution at 37 °C for 12 h. Then, 5 mL of PBS-containing collagenase was added, and the microneedles were fully dissolved at 37 °C for 30 min.

The sample preparation process in Figure 5c: 400 mg/mL GelMA solution, 200 mg/mL GelMA solution, a mixture of 250 μL of NDV allantoic fluid and 250 μL of 400 mg/mL GelMA solution, and collagenase solution. These samples were directly added into the experimental well. NDV allantoic fluid thawed at −80 °C was used as the positive control. PBS was used as the negative control. The steps of the hemagglutination test are set out in detail in the Supplementary Materials Table S2.

### 4.16. Statistical Analysis

Data analysis was conducted using GraphPad Prism version 9. In each group, at least three replicates were set up, and the results were presented in the form of mean ± standard deviation (SD). Differences of two groups and multiple groups were analyzed using Student’s *t*-test and one-way analysis-of-variance (ANOVA), respectively. Significant differences were presented as * *p* < 0.05, ** *p* < 0.01, *** *p* < 0.001, **** *p* < 0.0001.

## 5. Conclusions

In this study we successfully prepared GelMA hydrogel microneedles encapsulating the NDV. The microneedle have the swelling and degradation properties of hydrogel and excellent skin puncture performance, and the encapsulated NDV has tumor selective killing. Therefore, the GelMA hydrogel microneedles encapsulating NDV have excel-lent biocompatibility, safety, and tumor targeting. The GelMA hydrogel microneedles can effectively encapsulate and stabilize NDV in the gel system. However, NDV released by the delivery system was not detected in this study. Further experiments should be conducted to verify the release of NDV. Furthermore, different types of hydrogels or soluble matrix materials can be used to encapsulate NDV, and GelMA hydrogel microneedles can be designed as an in situ inflammatory niche to stimulate the body to produce a systemic anti-tumor immune response. In view of the limitations of the current delivery of oncolytic viruses using microneedles, we proposed corresponding solutions, including using freeze-drying technology to maintain the activity of the virus, using coated microneedles to increase the virus load, and adding sucrose, maltose, and other components to the matrix solution to enhance the stability of the virus. The implementation of these schemes has the potential to improve the activity, stability, and load of viruses loaded using microneedles. In conclusion, this study used GelMA hydrogel microneedles to deliver oncolytic NDV, providing a promising drug delivery scheme for oncolytic virus delivery.

## Data Availability

Data supporting the findings of this study are available from the corresponding author upon reasonable request.

## Supplementary Materials

The following supporting information can be downloaded at: https://www.mdpi.com/article/10.3390/ijms25042353/s1, supporting information is available from the MDPI online library or the authors.
