# Supplementary material for: Exploration of the Delivery of Oncolytic Newcastle Disease Virus by Gelatin Methacryloyl Microneedles
Source: Int J Mol Sci. 2024 Feb 16;25(4):2353. doi: 10.3390/ijms25042353 (PMC10888545; doi:10.3390/ijms25042353)
Supplement: Supplementary file 1 [file ijms-25-02353-s001.zip › ijms-2801883-supplementary.pdf]

Exploration of the Delivery of Oncolytic Newcastle Disease Virus by Gelatin Methacryloyl Microneedles

1. **Table S1. NDV TCID50 determination results.**

Dilution Degree	Number of Positive Holes	Number of Negative Holes	Cumulative Number of Positive Holes	Cumulative Number of Negative Holes	Percentage of Positive Cumulative Holes	Percentage of Positive Cumulative Holes (%)
10^{-1}	8	0	48	0	48/(48 + 0)	100
10^{-2}	8	0	40	0	40/(40 + 0)	100
10^{-3}	8	0	32	0	32/(32 + 0)	100
10^{-4}	8	0	24	0	24/(24 + 0)	100
10^{-5}	8	0	16	0	16/(16 + 0)	100
10^{-6}	8	0	8	0	8/(8 + 0)	100
10^{-7}	0	8	0	8	0/(0 + 8)	0
10^{-8}	0	8	0	16	0/(0 + 16)	0
10^{-9}	0	8	0	24	0/(0 + 24)	0
10^{-10}	0	8	0	32	0/(0 + 32)	0
10^{-11}	0	8	0	40	0/(0 + 40)	0
negative control	0	8	0	0	0/(0 + 48)	0

2. **Immunofluorescence staining steps**

The following were prepared: a 1% BSA solution, 1 × PBST solution, chicken anti-NDV primary antibody dilution, and FITC labeled sheep anti-chicken fluorescent secondary antibody dilution. DAPI solution was thawed at 4°C. A 96-well plate was obtained. The old culture medium was discarded and 100 μL PBS was added to each well. The plate was placed on a 100 rpm shaker for 5 min. Each well was washed three times and patted dry. Next, 50 μL of 4% paraformaldehyde solution was added to each well and placed in a 37°C 5% CO_2 incubator for 30 min. The paraformaldehyde was discarded and 100 μL PBST was added to each well. The plate was placed on a 100 rpm shaker for 5 min and each well was washed 3 times and patted dry. Next, 20 μL of chicken anti NDV primary antibody dilution was to each well and placed in a 37°C 5% CO_2 incubator for 30 min. The primary antibody diluent was discarded and 100 μL PBST was added to each well. The plate was placed on a 100 rpm shaker for 5 min, and each well was washed 3 times and patted dry. The following steps should be kept away from light： 20 μL FITC labeled sheep anti chicken fluorescent secondary antibody diluent was added into each well and placed in a 37°C 5% CO_2 incubator for 30 min. The secondary antibody diluent was discarded and 100 μL of PBST was added to each well and the plate was placed on a 100 rpm shaker for 5 min. Each well was washed 3 times and patted dry. DAPI solution 20 μL was added to each well and incubated at room temperature for 2 min to stain the nuclei. The DAPI solution was discarded and 100 μL PBS was added to each hole. The plate was placed on a 100 rpm shaker for 5 min, and each well was washed 3 times and patted dry, leaving 100 μL PBS in the hole for the last time.

3. Calculation formula of TCID50, PFU and MOI

The TCID50 of NDV was calculated according to the Reed-Muencha method as follows:

1) Positive cumulative hole ratio formula:

Percentage of positive cumulative holes (%) = cumulative number of positive holes / (cumulative number of positive holes + cumulative number of negative holes) × 100%

2) Distance ratio formula:

Distance ratio = - ((percentage of positive cumulative holes higher than 50% - 50%) / (percentage of positive cumulative holes higher than 50% - the percentage of positive cumulative holes lower than 50%)) × lg dilution factor

3) Formula of TCID50:

lg TCID50=lg Dilution ratio of positive cumulative wells above 50% + distance ratio ×difference between logarithm of dilution

4) PFU formula:

PFU=0.7 × TCID50

5) Formula for MOI value:

MOI value = (virus volume × PFU) / number of infected cells

4. Hemagglutination test steps

A 1% chicken red blood cell suspension was prepared: 5 mL of fresh chicken blood was collected and immediately added to the test tube containing 45 mL of sterile PBS, mixing it upside down gently to prevent coagulation. After centrifugation at 4000 rpm for 10 min, the supernatant was discarded, and the red blood cells were retained. Then 30 mL of sterile PBS to was added to red blood cells for washing followed by gentle mixing. After centrifugation at 4000rpm for 10 min, the supernatant was discarded and the step was repeated again. The remaining chicken red blood cells were resuspended with sterile PBS to prepare 1% chicken red blood cell suspension, and stored at 4°C for standby.

Hemagglutination test: a 96-well U-shaped plate was obtained and 50 μL PBS buffer was added to each hole. The collected sample solution was added to the first hole, mixed with a pipette gun, then 50 μL of solution were aspirated and added to the second hole, and the solution was diluted in multiple ratios in turn, discarding 50 μL of solution after mixing in the last hole. Then, 50 μL 1% chicken red blood cell suspension was added to each well, gently mixed, and the 96-well plate was placed at room temperature for 20 min.

Results: the 96-well plate was turned 90 degrees and placed vertically, and the red blood cell agglutination in the U-shaped hole was observed on a white background. If the red blood cells were at the bottom of the hole, the hemagglutination test was positive, indicating that the virus combined with the red blood cells; conversely, if the red blood cells flowed, the hemagglutination test was negative, indicating that there was no virus binding with the red blood cells. The maximum dilution of allantoic fluid with positive agglutination was used as the hemagglutination titer of the virus. A schematic diagram of the hemagglutination test is shown in Supplementary Table S2

5. Table S2. Schematic diagram of the hemagglutination test.

Number	1	2	3	4	5	6	7	8	9	10	11	12
Dilution multiple	2^1	2^2	2^3	2^4	2^5	2^6	2^7	2^8	2^9	2^{10}	2^{11}	2^{12}
PBS(μL)	50	50	50	50	50	50	50	50	50	50	50	50
Allantoic fluid(μL)	50	50	50	50	50	50	50	50	50	50	50	50
1% chicken red blood cell suspension(μL)	50	50	50	50	50	50	50	50	50	50	50	50
